# Estimation of the cardiovascular risk using world health organization/international society of hypertension risk prediction charts in Central Vietnam

**DOI:** 10.1371/journal.pone.0242666

**Published:** 2020-11-23

**Authors:** Ho Anh Hien, Nguyen Minh Tam, Vo Tam, Huynh Van Minh, Nguyen Phuong Hoa, Stefan Heytens, Anselme Derese, Dirk Devroey

**Affiliations:** 1 Department of Family Medicine, Hue University of Medicine and Pharmacy, Hue University, Hue, Vietnam; 2 Department of Public Health and Primary Care, Faculty of Medicine and Health Sciences, Ghent University, Ghent, Belgium; 3 Department of Internal Medicine, Hue University of Medicine and Pharmacy, Hue University, Hue, Vietnam; 4 Department of Family Medicine, Hanoi Medical University, Hanoi, Vietnam; 5 Department of Family Medicine and Chronic Care, Faculty of Medicine and Pharmacy, Vrije Universiteit Brussel, Brussels, Belgium; Johns Hopkins University, UNITED STATES

## Abstract

**Introduction:**

Cardiovascular disease (CVD) being the leading cause of the morbidity and mortality in Vietnam, the objective of this study was to estimate the total 10-year CVD risk among adults aged 40–69 years by utilizing World Health Organization/International Society of Hypertension (WHO/ISH) risk prediction charts in Central Vietnam.

**Materials and methods:**

In this cross-sectional study, multi-staged sampling was used to select 938 participants from a general population aged from 40 to 69. The CVD risk factors were then collected throughout the interviews with a standardized questionnaire, anthropometric measurements and a blood test. The cardiovascular risk was calculated using the WHO/ISH risk prediction charts.

**Results:**

According to the WHO/ISH charts, the proportion of moderate risk (10–20%) and high risk (>20%) among the surveyed participants were equal (5.1%). When “blood pressure of more than 160/100 mmHg” was applied, the proportion of moderate risk reduced to 2.3% while the high risk increased markedly to 12.8%. Those proportions were higher in men than in women (at 18.3% and 8.5% respectively, p-value <0.001, among the high-risk group), increasing with age. Male gender, smoking, ethnic minorities, hypertension and diabetes were associated with increased CVD risk.

**Conclusions:**

There was a high burden of CVD risk in Central Vietnam as assessed with the WHO/ISH risk prediction charts, especially in men and among the ethnic minorities. The use of WHO/ISH charts provided a feasible and affordable screening tool in estimating the cardiovascular risk in primary care settings.

## Introduction

The global number of deaths from CVD has increased by 12.5% during the past decade. CVD now accounts for approximately one third of all deaths worldwide, at more than 17.6 million deaths per year in 2016, a number that is expected to grow to more than 23.6 million by 2030. These changes have been driven by the population growth and aging populations, with the highest rate occurring in countries of South and East Asia because of their large and growing populations [1, 2]. In Vietnam, out of 520,000 total deaths, non-communicable diseases were estimated to account for 73%. CVD was estimated to occupy 33% of all deaths [3] and stroke was found to be the leading cause of death among them [4]. In addition, the morbidity and mortality of CVD in hospital settings were 9,0% and 18,6% respectively [5].

The rate of progression of atherosclerosis is influenced by cardiovascular risk factors namely tobacco use, an unhealthy diet, physical inactivity, hypertension, dyslipidaemia and diabetes [6]. In this light, the best approach should be using a total risk score for predicting the cardiovascular risk, such as in the Framingham Risk Score Model for the American, and SCORE (Systematic COronary Risk Evaluation) for the European [7, 8]. These scores, however, were based upon studies conducted in high-income areas, so they might not be suitable for use in low-resource settings. In addition, some models had been developed to predict the CVD risk in Thailand, China, Japan, and other Asian populations [9–12]. Some inputs to the models included sex, age, smoking status, information on diabetes, stage of hypertension as well as systolic blood pressure, total cholesterol, and high-density lipoprotein cholesterol levels. Particularly, all of these models required figures for the last two factors, the tests of which were difficult to be conducted at the community health centers in Central Vietnam.

Sets of regional risk prediction charts were therefore developed by World Health Organization and the International Society of Hypertension (WHO/ISH) for each of the fourteen WHO sub-regions based on fewer risk factors that can be assessed by physicians and non-physician healthcare workers in primary care settings (with or without blood cholesterol) [6, 13]. There was one chart that calculated the total risk of developing fatal and non-fatal CVDs in the following 10 years among those whose CVDs were asymptomatic. The chart estimated the population distribution of the total CVD risk to assess the total preventive needs and to improve the efficiency of cardiovascular prevention in areas of limited resources. In addition, it helped to identify the high-risk people in need of pharmacological treatment and lifestyle behavioral changes [14, 15].

The CVD situation in Central Vietnam has not been well-documented during the last 10 years. This however is important for Vietnamese health planners in designing effective intervention strategies. We had already published a paper on the prevalence, awareness, treatment and control of hypertension as an important single risk factor of CVD [16]. In the present paper, we studied the combined influence of multiple risk factors for CVD risk prediction, given the known interaction between those many different factors. This approach would especially be helpful in communities with limited resources, of which there reported to be many in Central Vietnam, since it could help focus the medication to be prescribed, and hence save money [6, 14, 15, 17]. In this paper, we used the WHO/ISH risk prediction charts to calculate the total 10-year cardiovascular risk among adults aged 40–69 in Central Vietnam.

## Materials and methods

### Study design and population

A cross-sectional study was conducted from April to October 2015 to estimate the cardiovascular risk among adults aged 40–69 years, using the WHO/ISH risk charts with every individual that had a blood pressure of above 160/100 mmHg being considered “high risk” [6, 18]. The sampling method, data collection procedures and variable measurements were clearly delineated in our previous study [16]. In brief, the research data were gathered in a typical province of Central Vietnam called Thua Thien-Hue during a population-based survey project. One urban district, one low-lying district and one mountainous district were selected for the sake of a sample representing the diversity of this province. In each district, two communes were randomly selected. In each of these communes, every 10^th^ person aged 40 to 69 was chosen from the 2013 local governmental household register, leading to 1,200 participants selected across the whole province in question.

The selected persons were invited to visit one of the six local community health centers in the morning on dates specifically indicated in the invitation letter. A well-trained team of 15 doctors and medical students were in charge of the interviews and physical examinations using a standardized questionnaire based on the WHO’s Stepwise approach [19]. The questionnaire comprised questions on their demographics, lifestyle behaviours and history of hypertension, diabetes and CVDs. The examination list included measuring their blood pressure, capillary blood glucose, along with anthropometric measurement.

The research participants’ *blood pressure* was measured in a sitting position at least twice with automatic sphygmomanometers. They were asked to take a rest of 15 minutes before the first measurement, and the second one took place about 5 minutes later. If there was a difference of more than 10 mmHg between these two measurements, a third measurement was performed. The average of the last 2 measurements was calculated and used in the analysis [20].

*Hypertension* was defined as a systolic blood pressure value of 140 mmHg or more and/ or a diastolic blood pressure value of 90 mmHg. Hypertension was classified into five stages (Optimal and normal, High normal, Stage-1 hypertension, Stage-2 hypertension and Stage-3 hypertension) according to the Seventh Joint National Committee on Hypertension Classification [20, 21].

Fasting capillary *blood glucose* was measured by means of a glucometer (One Touch Ultra 2, Lifescan, US) using the standard protocol, provided by the manufacturer. In addition to the use of insulin or oral hypoglycemic drugs at the time of our survey, which indicated that the participant had diabetes, we could determine the 3 diabetes stages based upon the fasting capillary blood glucose as follows: ≥7.0 mmol/l on 2 different days for Diabetes, 6.1–6.9 mmol/l for Pre-diabetes, and <6.1 mmol/l for No-diabetes [22].

A *current smoker* was defined as a person currently consuming tobacco products or having quit smoking for less than 12 months. *Body Mass Index* (BMI) was calculated as weight (kg) per height squared (m^2^). At the time of our survey, *overweight and obesity* was defined by WHO Regional Office for Western Pacific BMI from 23 kg/m^2^ [23]. *Abdominal obesity* for the Asian population included those persons whose waist hip ratio valued ≥0.9 for men or ≥0.8 for women [24]. People with fewer than 600 MET/min/week of physical activity were categorized as *physical inactivity*, or low level of physical activity [19].

### Calculating the total CVD risk using WHO/ISH risk prediction charts

The total CVD risk of the sampled individuals was calculated using the WHO/ISH risk assessment charts for the Western Pacific B sub-region (WPR B) without blood cholesterol [6, 13]. No-cholesterol charts were employed because of the resource limitations. The no-cholesterol risk estimation was based on gender (male/female), age group (40–49, 50–59, and 60–69), systolic blood pressure level (120–139, 140–159, 160–179 and ≥180 mmHg), current smoking status (non-smoker or smoker) and diabetes status (yes or no). The WHO-ISH prediction charts were applied to estimate the 10-year risk of a fatal or non-fatal major CVD event (myocardial infarction or stroke) being divided into four categories: ≤10% (low), 10–19% (moderate), 20–29% (high), and ≥30% (very high) in people without established CVDs [6, 19]. In this study, the three risk categories for 10-year total risk of a fatal or non-fatal CVD event included <10% classified as “low risk”, 10- <20% as “moderate risk”, and ≥20% as “high risk” [17, 18].

The CVD risk among adults aged more than 40 years was estimated utilizing the risk chart alone. In parallel with this, we added to the chart the criterion of “blood pressure >160/100 mmHg” and recalculated the CVD risk, as it was stated in the practice notes for clinicians in WHO/ISH Guidelines that those with blood pressure consistently higher than 160/100 mmHg at measurement should be considered high-risk regardless of their risk calculations [6].

### Ethical approval

The research proposal was approved by Hanoi Medical University, with Decision number 3680/QD-DHYHN, and health departments of the three districts involved. The study was explained to all respondents willing to participate in it, and all participants granted their consent before participating in the study. All participants had the right to withdraw from the study at any time. High-risk patients were advised to consult their community health center or district hospital for a follow-up.

### Statistical analysis

Frequency distributions and percentages were computed for all the variables. All statistical results were based on two-sided tests and p-value <0.05 was considered to represent statistical significance. Percentages and 95 confidence intervals were computed, and a Chi-square test was used for categorical variables. Epidata entry (version 3.1; Epidata Association, Denmark) was used to record the study data. Both descriptive and analytical statistical analyses were carried out using IBM SPSS (version 23.0; IBM Corp., Armonk, New York, USA).

## Results

The participant response rate was 81.9%, with a total number of 983 people turning up at the local community health centers. After excluding 14 records with missing information on hypertension risk factors and 31 records with history of cardiovascular diseases, 938 participants were included for analysis.

Table 1 reveals the different socio-demographic variables according to the gender of the study participants. 56.3% of the participants were female, rural residence accounted for 66.0% of the total participants, and approximately one third of the surveyed population belonged to ethnic minorities. At the present time of the study, the ratio of smoking men to smoking women in question was about 3:1 (53.9% vs. 17.2%, p <0.001). Excessive alcohol consumption was also higher in men than in women (16.6% vs. 0.9%, p <0.001). However, the prevalence of physical inactivity was similar between both genders. The proportion of overweight, including obesity and abdominal obesity, were higher in women than in men (39.6% vs. 32.7%; 70.3% vs. 37.3%, p <0.001). The prevalence of hypertension was 42.0%; this percentage was higher in men than in women at all stages of hypertension. The prevalence of diabetes accounted for 5.0%; there was no significant difference between the two genders.

**Table 1:** Characteristics of the study population aged 40–69 years by gender.

Characteristics	Men	Women	Total	p
	n (%)	n (%)	n (%)	
***Age Group***	410 (43.7)	528 (56.3)	938 (%)	0.01
*40–49*	111 (27.1)	145 (27.5)	256 (27.3)	
*50–59*	130 (31.7)	211 (40.0)	341 (36.4)	
*60–69*	169 (41.2)	172 (32.5)	341 (36.4)	
***Residence***				0.37
*Urban*	133 (32.4)	186 (35.2)	319 (34.0)	
*Rural*	277 (67.6)	342 (64.8)	619 (66.0)	
***Ethnicity***				0.84
*Ethnic Majority (Kinh)*	288 (70.2)	374 (70.8)	662 (70.8)	
*Ethnic Minorities*	122 (29.8)	154 (29.2)	276 (29.2)	
***Educational Level***				<0.001
*Primary school or under*	184 (44.9)	373 (70.6)	557 (59.4)	
*Middle or High school*	196 (47.8)	137 (25.9)	333 (35.5)	
*College or University*	30 (7.3)	18 (3.4)	48 (5.1)	
***Occupation***				<0.001
*Manual Worker*	213 (52.0)	211 (40.0)	424 (45.2)	
*Governmental Staff*	103 (25.1)	53 (10.0)	156 (16.6)	
*Other Occupations*	94 (22.9)	264 (50.0)	358 (38.2)	
***Current Smoking Status***				<0.001
*Yes*	221 (53.9)	91 (17.2)	312 (33.3)	
*No*	189 (46.1)	437 (82.8)	626 (66.7)	
***Excessive Alcohol Consumption***				<0.001
*Yes*	68 (16.6)	5 (0.9)	73 (7.8)	
*No*	342 (83.4)	523 (99.1)	865 (92.4)	
***Physical Activity Level***				0.39
*Low*	41 (10.0)	62 (11.7)	103 (11.0)	
*Moderate*	69 (16.8)	82 (15.5)	151 (16.1)	
*High*	300 (73.2)	384 (72.7)	684 (72.9)	
***Body Mass Index***				0.03
*Underweight*	65 (15.9)	54 (10.2)	119 (12.7)	
*Normal Weight*	211 (51.5)	265 (50.2)	476 (50.7)	
*Overweight*, *Obesity*	138 (32.7)	201 (39.6)	343 (36.5)	
***Abdominal Obesity***				<0.001
*Yes*	154 (37.6)	371 (70.3)	525 (56.0)	
*No*	256 (62.4)	157 (29.7)	413 (44.0)	
***Classification of Blood Pressure***				<0.001
*Optimal & Normal*	167 (40.7)	318 (60.2)	485 (51.7)	
*High normal*	74 (18.0)	79 (15.0)	153 (26.3)	
*Stage-1 Hypertension*	96 (23.4)	86 (16.3)	182 (19.4)	
*Stage-2 Hypertension*	39 (9.5)	30 (5.7)	69 (7.4)	
*Stage-3 Hypertension*	34 (8.3)	15 (2.8)	49 (5.2)	
***Blood Glucose***				0.16
*Diabetes*	22 (5.4)	24 (4.5)	47 (4.9)	
*Pre-diabetes*	42 (10.2)	37 (7.0)	79 (8.4)	
*No-diabetes*	346 (84,4)	467 (88.4)	813 (86.7)	

Table 2 demonstrates the distribution of CVD risk based on the combination of distinctive characteristics. Using the WHO/ISH charts alone, 5.1% participants were predicted to have a high risk (>20%) of a CVD event in 10 years. CVD risk was higher in men than in women (8.6% vs. 2.5%, p <0.01). The prevalence of moderate and high CVD risk in men was higher than in women. When “blood pressure of more than 160/100 mmHg” was applied, the participants with low and moderate risk reduced to 84.9% and 2.3% correspondingly (76.8% and 4.9% in men; 91.1% and 0.4% in women). However, it was found that 12.8% participants (18.3% in men and 8.5% in women) had some CVD-related outcome.

**Table 2:** Risk of CVD with different inclusion criteria for the risk factors in adults aged 40–69.

Inclusion criteria	Gender	Ten-Year CVD Risk			p
		Low n (%)	Moderate n (%)	High n (%)	
**WHO/ISH Chart alone**	Men	332 (81.0)	43 (10.5)	35 (8.5)	<0.001
	Women	510 (96.6)	5 (0.9)	13 (2.5)	
	Total	842 (89.8)	48 (5.1)	48 (5.1)	
**WHO/ISH Chart + BP >160/100**	Men	315 (76.8)	20 (4.9)	75 (18.3)	<0.001
	Women	481 (91.1)	2 (0.4)	45 (8.5)	
	Total	796 (84.9)	22 (2.3)	120 (12.8)	

Table 3 compares the distribution of CVD risk groups in different age categories. The total percentage of high CVD risk was 15.5% whereas that of low CVD risk was almost five times higher, at 78.0%. As far as male participants were concerned, the prevalence of high CVD risk increased slightly from 14.4% for the 40–49 age group to 16.7% for the 50–59 age group and then rose sharply to 22.5% in the 60–69 age group. When it came to their female counterparts, the high-risk proportion decreased from 10.4% among women aged 50–59 to merely 8.7% among the oldest group surveyed. There was quite a big difference between the percentages of men and women of the age group 60–69 in the moderate- and high-risk groups.

**Table 3:** CVD risk categories among adults aged 40–69 by gender and age group.

Gender	Age group	Cardiovascular Risk			Cardiovascular Risk		
		(Chart alone)			(Chart + BP >160/100)		
		Low	Moderate	High	Low	Moderate	High
		n (%)	n (%)	n (%)	n (%)	n (%)	n (%)
**Men**	40–49 (n = 111)	107	4	0	95	0	16
		(96.4)	(3.6)		(85.6)		(14.4)
	50–59 (n = 130)	114	4	12	109	0	21
		(87.7)	(3.1)	(9.2)	(83.3)		(16.7)
	60–69 (n = 169)	111	35	23	111	20	75
		(65.7)	(20.7)	(13.6)	(65,7)	(11.8)	(22.5)
**p**		<0.001			<0.001		
**Women**	40–49 (n = 145)	143	2	0	137	0	8
		(98.6)	(1.4)		(94,5)		(5.5)
	50–59 (n = 211)	201	0	10	189	0	22
		(95.3)		(4.7)	(89.6)		(10.4)
	60–69 (n = 172)	166	3	3	155	2	15
		(96.5)	(1.7)	(1.7)	(90.1)	(1.2)	(8.7)
**p**		0.018			0.14		
**Total**	40–49 (n = 256)	250	6	0	232 (90.6)	0	24 (9.4)
		(97.7)	(2.3)				
	50–59 (n = 341)	315	4	22	298 (87.4)	0	43 (12.6)
		(92.4)	(1.2)	(6.5)			
	60–69 (n = 341)	277	38	26	266	22	53
		(81.2)	(11.1)	(7.6)	(78.0)	(2.3)	(15.5)
**p**		<0.001			<0.001		

Table 4 presents how significantly the prevalence of men, ethnic minority residents, those with current smoking status, hypertension and diabetes in the high-risk CVD groups were higher than those in the low-risk ones. There was no significant difference in terms of residence, educational level, excessive alcohol consumption, physical inactivity, overweight-obesity, and abdominal obesity. The proportion of hypertension awareness and treatment in the high-risk groups were relatively low. Patients with controlled hypertension were classified as low-risk.

**Table 4:** Prevalence of CVD risk factors by CVD risk category.

Inclusion criteria	CVD Risk			p
	(Chart + BP >160/100)			
	Low	Moderate	High	
	(<10%)	(10–20%)	(>20%)	
Gender (Men)	315 (39.6)	20 (90.9)	75 (62.5)	< 0.001
Age (60–69)	266 (33.4)	22 (100)	53 (44.2)	< 0.001
Current Smoking (Yes)	245 (30.8)	21 (95.5)	46 (38.3)	< 0.001
Diabetes (Yes)	32 (4.0)	5 (22.7)	9 (7.5)	0.003
Hypertension (Yes)	271 (34.0)	21 (95.5)	120 (100)	< 0.001
Ethnicity (Ethnic Minority)	221 (27.8)	6 (27.3)	49 (40.8)	0.008
Residence (Rural)	519 (65.2)	16 (72.7)	84 (70.4)	0.22
Education (Primary School or Under)	479 (60.2)	14 (63.6)	64 (53.3)	0.017
Excessive Alcohol Consumption (Yes)	58 (7.3)	0	15 (12.5)	0.18
Physical Inactivity	85 (10.7)	2 (9.1)	16 (13.3)	0.48
Overweight, Obesity (Yes)	287 (36.1)	6 (27.3)	50 (41.7)	0.44
Abdominal Obesity (Yes)	451 (56.7)	9 (40.9)	65 (54.2)	0.31
Hypertension Awareness	182 (67.2)	14 (66.7)	76 (63.3)	0.5
Hypertension Treatment	84 (46.2)	5 (35.7)	43 (56.6)	0.26
Hypertension Control	50 (59.5)	0	0	<0.001

p*: p-value when we compared between low-risk groups and moderate-, high-risk groups.

## Discussion

Our study aimed at estimating the total 10-year CVD risk among a population representative of Central Vietnam. Utilizing the WHO/ISH risk prediction chart alone, 10.2% of the population surveyed was found to be at moderate and high risk (≥10%). This proportion was consistent with findings from a study in the rural mountainous areas in the North of Vietnam (2012) [25], in which the prevalence of (≥10%) CVD risk according to the Chinese Multiple provincial cohort study (MCCS) and the Asian model was 11.7% and 9.07% respectively. The percentage found in the year 2012, however, increased to 28.8% when the Framingham model was applied [25]. When the factor of “blood pressure >160/100 mmHg” was added to the WHO/ISH prediction risk chart in the present study, the total prevalence of moderate and high risk was 15.1%, higher than the figures for the MCCS and Asian models but lower than the figure for the Framingham model. As had been suggested by the literature, the use of the Framingham model in an Asian or European context was linked to overestimated risk percentages because of the CVD lower rates [10, 26], and this remark was confirmed in another study also conducted in Northern Vietnam but in the year 2009, where the Framingham model showed similar high results of 27% [27].

The results of our study were also consistent with findings from some other Asian studies using the WHO/ISH prediction charts (India 4.3%, Mongolia 6.0%, and Nepal 4.9%), but higher than those of other studies (China 1.1%, Iran 1.7%, Sri Lanka 2.2%, Cambodia 1.3%, and Malaysia 2.3%) [14, 18]. The prevalence of high risk doubled, at 12.8%, after we added “blood pressure >160/100 mmHg” to the WHO/ISH prediction risk chart. This proportion was similar to that of India (10.2%) and Nepal (11%) but again higher than that of Cambodia (4.8%) and Malaysia (7.7%) [14, 17, 18].

These consistent results justified the practical value of the WHO/ISH model, which was selected to estimate the total CVD risk in the present research. An added advantage of this model was that it did not include any blood cholesterol or blood high-density lipoprotein cholesterol tests as in the other prediction models (MCCS model, Asian model, Framingham model or SCORE model) [6–8, 10, 12], which reduced the medical cost and equipment in low-resource primary care settings in Vietnam. In the meantime, the primary healthcare providers could find it more feasible to provide medical information of CVD risk for the patients and also to estimate the total CVD risk population in their communities [14, 17]. The WHO/ISH prediction chart plus other criteria such as “blood pressure >160/100 mmHg”, on the other hand, was able to provide further information about the CVD risk in the community in case the WHO/ISH chart alone underestimated the total CVD risk due to the lack of some risk factors in the model such as blood cholesterol or the family history of CVD and obesity, especially among the patients with antihypertensive medication [6].

Using the WHO/ISH prediction charts, a higher proportion of men had moderate or high CVD risk than women (Table 2). This trend was similar to previous studies in Vietnam and other countries in Asia like India, Nepal, Cambodia, or Malaysia [14, 17, 18]. This was easy to understand because the prevalence of hypertension, diabetes, and smoking was higher in men than in women. The risk among both men and women increased with age. There was a comparatively big difference between the percentages of moderate risk and the high risk of men and women aged 60–69. This mainly stemmed from the fact that there was a protective effect on the women’s estrogen level [28, 29] and that the prevalence of hypertension and smoking were higher in men than in women in the 60–69 age group.

As had been expected, the research participants’ CVD risk was significantly associated with their gender, age, smoking status, hypertension level, and ethnicity. The ethnic minorities in our study mostly lived in remote and rural areas with restricted healthcare resources. The prevalence of hypertension, hypertension control, diabetes, and smoking among ethnic minorities were higher than among Kinh people—the ethnic majority [16]. The prevalence of overweight, abdominal obesity, physical inactivity, and excessive alcohol consumption were highest in the high-risk CVD group. This finding was consistent with previous studies in Malaysia (2013), Mongolia (2013), and India (2015) [6, 17, 18], which meant that the more CVD risk factors the patients had, the more likely they were to be put in the high-risk CVD group. Unfortunately, there was no significant statistical association between the CVD risk and overweight, abdominal obesity, physical inactivity, or excessive alcohol consumption.

The proportion of hypertension awareness and treatment were low in the high-risk group. In the meantime, the patients with controlled hypertension were classified as low-risk (Table 4). It was suggested by our study that the most important determinant to decrease CVD risk was to control the hypertension rates.

High prevalence of hypertension has long been one of the major public health problems in Central Vietnam; however, low prevalence has still been attached to the treatment and control of hypertension [16, 30–32]. Only 12.8% of the research population needed medication when we used the approach of total CVD risk (≥20%), while according to our previous study, which adopted the single risk factor approach, there was as much as 44.8% of the population in need of medication [16]. Therefore, over-prescription of the medication would be prevented, especially in low-resourced settings, if we employed the total CVD risk approach.

### Limitations

Admittedly, using the WHO/ISH prediction risk charts in a cross-sectional study had some limitations. On the one hand, the results of the study might underestimate the total CVD risk because they did not include the individuals’ anti-hypertensive therapy, blood cholesterol, family history of CVD and obesity. On the other hand, the blood pressure measured on single visits in this study could overestimate the prevalence of hypertension and the proportion of the population with blood pressure persistently higher than 160/100 mmHg [6, 17].

### Implications

The research results offered some practical implications as follows: The total CVD risk statistics in this study, first of all, could provide important information about how to help the local health planners to implement effective intervention measures to reduce the CVD burden. The primary healthcare providers in restricted-resource settings could also make full use of the WHO/ISH prediction charts in their efforts to provide a more feasible and affordable measurement of the total CVD risk for individual patients.

## Conclusions

There was a high burden of CVD risk in Central Vietnam as assessed with the WHO/ISH risk prediction charts, especially in men and among the ethnic minorities. Gender, age, ethnicity, smoking, hypertension and diabetes were associated with CVD risk. Almost all patients with controlled hypertension belonged to the low-risk CVD groups. The application of WHO/ISH charts provided a feasible and affordable screening tool for estimating the total cardiovascular risk in primary care settings. More effective solutions would be needed to reduce the CVD burden in Central Vietnam.

## Supporting information

S1 File Data CVD risk in central vietnam. (SAV)
