# Correction: Estimation of the cardiovascular risk using world health organization/international society of hypertension risk prediction charts in Central Vietnam

**DOI:** 10.1371/journal.pone.0261099

**Published:** 2021-12-02

**Authors:** Ho Anh Hien, Nguyen Minh Tam, Vo Tam, Huynh Van Minh, Nguyen Phuong Hoa, Stefan Heytens, Anselme Derese, Dirk Devroey

The authors are listed out of order. Please view the correct author order, affiliations, and citation here:

Ho Anh Hien^1,2*^, Nguyen Minh Tam^1*^, Vo Tam^3^, Huynh Van Minh^3^, Nguyen Phuong Hoa^4^, Stefan Heytens^5^, Anselme Derese^5^, Dirk Devroey^2^

**1** Department of Family Medicine, Hue University of Medicine and Pharmacy, Hue University, Hue, Vietnam

**2** Department of Family Medicine and Chronic Care, Faculty of Medicine and Pharmacy, Vrije Universiteit Brussel, Brussels, Belgium

**3** Department of Internal Medicine, Hue University of Medicine and Pharmacy, Hue University, Hue, Vietnam

**4** Department of Family Medicine, Hanoi Medical University, Hanoi, Vietnam

**5** Department of Public Health and Primary Care, Faculty of Medicine and Health Sciences, Ghent University, Ghent, Belgium
